# Neuroanatomical Moderators of the Impact of Mild Behavioral Impairment on Cognition

**DOI:** 10.1093/geroni/igab046.2631

**Published:** 2021-12-17

**Authors:** Hillary Rouse, Brent Small

**Affiliations:** 1 SiteRx / University of South Florida, TAMPA, Florida, United States; 2 University of South Florida, Tampa, Florida, United States

## Abstract

Older adults with mild behavioral impairment (MBI), or the presence of late-life neuropsychiatric symptoms, have a unique cognitive phenotype. However, the neural correlates associated with MBI-related cognitive changes is not well understood. The goal of this study is to examine if specific regions of the brain moderate the relationship between the presence of MBI and performance on tasks of cognition. Data from the National Alzheimer’s Coordinating Center was utilized for this study. Participants (N=1,451) were included in our analyses if they were cognitively healthy or had mild cognitive impairment (MCI). Multiple domains of cognitive performance were evaluated. The neuroanatomical regions included hippocampus, caudal anterior cingulate (ACC), rostral ACC, entorhinal, and parahippocampal gray matter volume; and caudal ACC, rostral ACC, entorhinal, and parahippocampal mean cortical thickness. Hippocampal, entorhinal, and parahippocampal cortical gray matter volume moderated the relationship between MBI and performance on tasks of episodic memory. Left rostral ACC cortical gray matter volume and entorhinal and parahippocampal mean cortical thickness moderated the relationship between MBI and performance on language tasks. Hippocampi cortical gray matter volume also moderated the relationship between MBI and performance on processing speed tasks. Persons with smaller brain sizes in these areas were more negatively affected in these cognitive domains if they had MBI. These results suggest that the association between smaller brain volumes and cognition was stronger among persons with MBI. These findings suggest that older adults with MBI may perform worse on these tasks due to neurodegeneration that is present.

